# Unraveling T cell exhaustion in the immune microenvironment of osteosarcoma via single-cell RNA transcriptome

**DOI:** 10.1007/s00262-023-03585-2

**Published:** 2024-01-27

**Authors:** Debin Cheng, Zhao Zhang, Dong Liu, Zhenzhou Mi, Weidong Tao, Jun Fu, Hongbin Fan

**Affiliations:** Department of Orthopaedic Surgery, Xi-Jing Hospital, The Fourth Military Medical University, Xi’an, 710032 China

**Keywords:** Osteosarcoma, Single-cell RNA sequencing, T cell exhaustion, Tumor immune microenvironment, Prognosis

## Abstract

**Abstract:**

Osteosarcoma (OS) represents a profoundly invasive malignancy of the skeletal system. T cell exhaustion (Tex) is known to facilitate immunosuppression and tumor progression, but its role in OS remains unclear. In this study, single-cell RNA sequencing data was employed to identify exhausted T cells within the tumor immune microenvironment (TIME) of OS. We found that exhausted T cells exhibited substantial infiltration in OS samples. Pseudotime trajectory analysis revealed a progressive increase in the expression of various Tex marker genes, including PDCD1, CTLA4, LAG3, ENTPD1, and HAVCR2 in OS. GSVA showed that apoptosis, fatty acid metabolism, xenobiotic metabolism, and the interferon pathway were significantly activated in exhausted T cells in OS. Subsequently, a prognostic model was constructed using two Tex-specific genes, MYC and FCGR2B, which exhibited exceptional prognostic accuracy in two independent cohorts. Drug sensitivity analysis revealed that OS patients with a low Tex risk were responsive to Dasatinib and Pazopanib. Finally, immunohistochemistry verified that *MYC* and *FCGR2B* were significantly upregulated in OS tissues compared with adjacent tissues. This study investigates the role of Tex within the TIME of OS, and offers novel insights into the mechanisms underlying disease progression as well as the potential treatment strategies for OS.

**Graphic Abstract:**

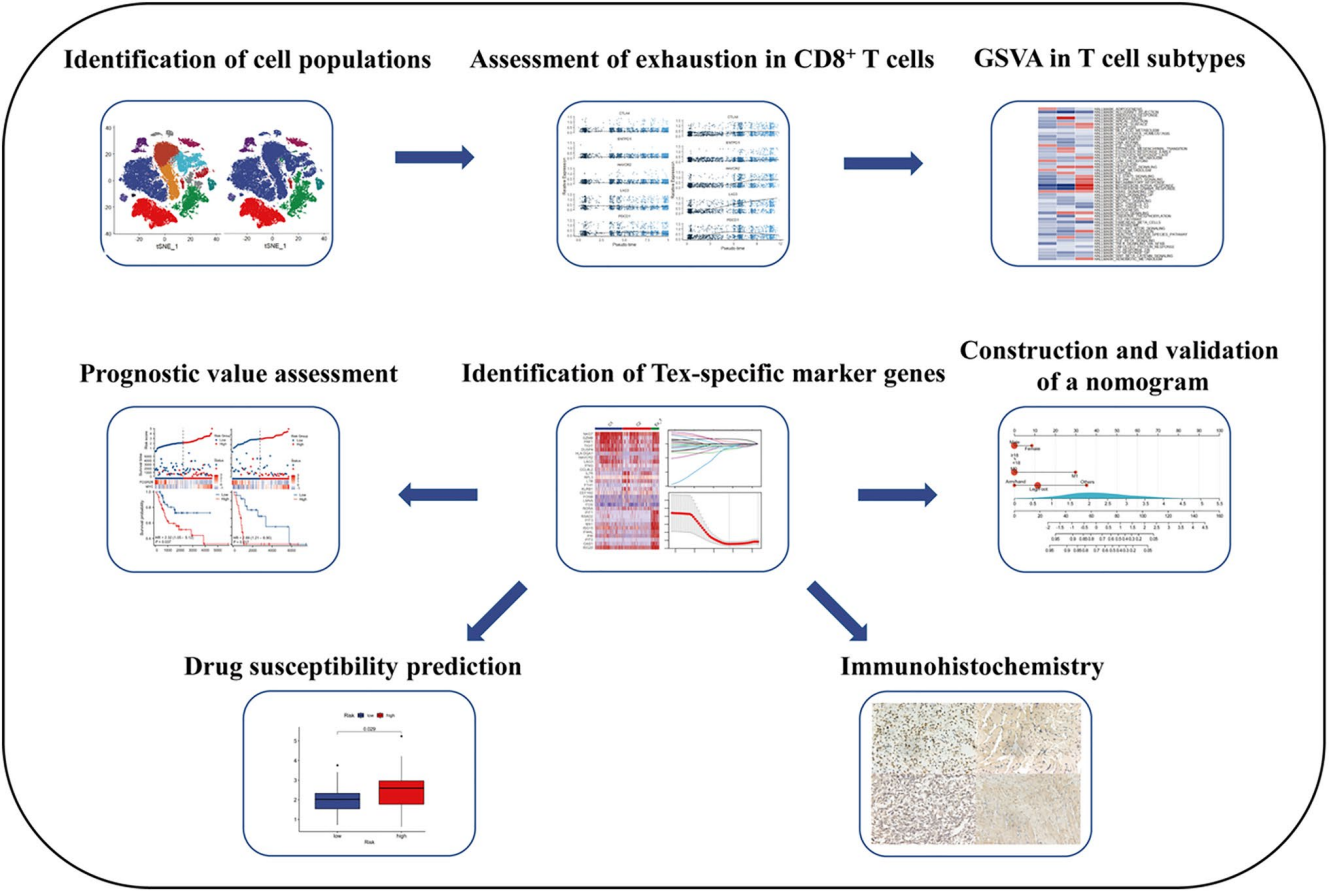

**Supplementary Information:**

The online version contains supplementary material available at 10.1007/s00262-023-03585-2.

## Introduction

Osteosarcoma (OS) is associated with malignant, highly aggressive and high heterogeneity tumors, which can seriously endanger the health and physical function of children and juveniles [1]. Despite various treatments for OS, including surgical resection, chemotherapy, immunotherapy, and targeted therapies, it continues to have a poor prognosis [2]. The pathophysiology of OS remains elusive; however, several studies have demonstrated a strong correlation between its pathogenesis and genetic characteristics [3]. Simultaneously, the intricate molecular heterogeneity and consequential functional perturbations within the tumor immune microenvironment (TIME) facilitate the tumor progression and emergence of chemoradiation resistance [4]. Hence, understanding the intricate TIME is important for discovering novel therapeutic targets.

The T cells residing within the TIME play a central role in cancer immune surveillance by impeding tumor progression. Intracellular pathogens and malignant cells are countered and eradicated by the influx of immune cells, most notably the CD8^+^ cytotoxic T lymphocytes, which directly kill tumor cells, thus forming the cornerstone of cancer immunotherapy [5]. A reduction in the number or functionality of CD8^+^ T cells within the host portends a decline in antitumor immunity, thereby increasing the risk of neoplastic growth and metastasis [6]. However, as the cancer progresses, the T cells that infiltrate the TIME may experience a late-stage exhaustion due to sustained stimulation by tumor antigens—a phenomenon called T cell exhaustion (Tex), which decreases the number or functionality of effector T cells, thereby enabling tumor immune evasion and progression [7]. Exhausted T cells (ExTs) isolated from advanced tumors exhibit the characteristics of tumor-infiltrating lymphocytes: they fail to secrete effector cytokines or cytotoxic molecules in response to tumor cells that express multiple inhibitory receptors [8]. In contrast, exhausted CD8^+^ T cells display a plethora of hallmark features to functional effector cells and memory T cells, such as a gradual loss of effector function, sustained upregulation of multiple co-inhibitory receptors, and changes in epigenetic regulation and metabolism [9]. Mounting evidence suggests that co-inhibitory checkpoint molecules, including programmed cell death-1 (PD-1), cytotoxic T lymphocyte-associated protein-4 (CTLA-4), and lymphocyte-activation gene-3 (LAG-3), are involved in the development of Tex [10]. Reversal of Tex within the TIME may represent a feasible strategy for controlling cancer progression. However, the precise contribution of Tex to the pathogenesis and progression of OS remains ambiguous, and understanding its role clearly will be crucial in identifying novel therapeutic targets and prognostic biomarkers for OS, which may improve clinical management and decision-making.

Conventional transcriptomic approaches fail to reliably characterize the complex TIME of OS because they investigate overall gene transcription in tumor samples, thus lacking the adequate resolution to identify specific cell types. The advent of single-cell genome sequencing has enabled the determination of rare cellular subsets and corresponding functional changes in the TIME [11]. In this study, we have integrated single-cell and bulk RNA-seq data to explore the role of Tex in the TIME of OS. We have also developed and validated a prognostic predicting model based on Tex-associated biomarkers, while predicting target drugs for OS patients. These findings shed light on the oncogenesis and progression of OS as well as its potential therapeutic strategy.

## Materials and methods

### Datasets utilized for analysis and preprocessing of the data

Single-cell RNA sequencing (scRNA-seq) datasets GSE169396 and GSE162454 were obtained from the Gene Expression Omnibus (GEO) database. GSE169396 harbored four health human bone tissues, while GSE162454 comprised six specimens obtained from osteosarcoma (OS) patients. The Seurat package was employed to manage both datasets. Additionally, bulk RNA sequencing (bulk RNA-seq) data and associated clinical details from the TARGET dataset and GSE21257 database were used as training and validation sets. The clinical characteristics of the OS patients from the TARGET dataset and GSE21257 database are presented in Table S1. For quality control purposes, genes expressed in at least five cells were retained, while cells exhibiting either less than 250 genes or more than 5000 genes were eliminated. Furthermore, cells exhibiting more than 10% mitochondrial reads were excluded. The NormalizeData and ScaleData functions were applied to standardize and scale the gene expression matrix, respectively. The top 3000 highly variable genes were identified using the FindVariableFeatures function, which served as input for principal component analysis (PCA). Batch effects of ten samples were corrected using the R package "Harmony". Following this, the FindNeighbors and FindClusters (resolution = 0.2) functions were executed to detect cell clusters. Then, the RunTSNE function was executed to achieve further dimensional reduction for cluster visualization. Additionally, T cell expression matrix was extracted and reclustered using the FindClusters (resolution = 0.6) function. Subsequently, CD8^+^ T cells were identified via recognized marker genes.

### Identification of cell populations and assessment of exhaustion in CD8^+^ T cells

The cell clusters in the TIME were annotated based on the well-established cell-specific markers from the previous literature [12, 13]. The CD8^+^ T cells were identified using CD2, CD3D, CD3E, CD3G, CD8A, and CD8B markers. The DotPlot function and ggbeeswarm package were employed to graphically depict the expression of marker genes in each cluster. The prop.table function was used to determine the proportions of CD8+ T cell subtypes with the stat_compare_means function to analyze the significant difference by t test. To calculate the exhaustion scores, AddModuleScore function was utilized by taking the average expression of five exhaustion-associated genes (CTLA4, PDCD1, ENTPD1, LAG3, HAVCR2) across all CD8^+^ T cells of normal bone and OS samples. Then, the exhaustion score of each cell was visualized in t-distributed Stochastic Neighbor Embedding (tSNE) plots. Furthermore, the VlnPlot function was used to compare exhaustion scores between normal bone and OS, while t test was conducted to determine the significance of exhaustion score in two groups.

### Pseudotime trajectories analysis CD8^+^ T cells

The analysis of pseudotime trajectories CD8^+^ T cells were conducted using the Monocle package (version 2.26.0) in R. Plot_cell_trajectory function was employed to represent the differentiation trajectory. To ascertain the expression levels of exhaustion-associated genes along the trajectory, the Plot_genes_in_pseudotime function was utilized. Furthermore, identification of cluster-specific genes was achieved through the implementation of the FindAllMarkers function. The expression levels of Top 15 genes were then depicted in a pseudotime heatmap using the plot_pseudotime_heatmap function.

### Gene set variation analysis (GSVA) in CD8^+^ T cell subtypes

We procured hallmark gene sets from the Molecular Signatures Database that encapsulate well-defined biological states, processes, or tumorigenesis. The differential signatures of cellular pathways in CD8^+^ T cell subtypes were ascertained by employing the GSVA package in R. The GSVA heatmap was generated by hepheatmap package.

### Identification of Tex-specific marker genes and screening of prognostic genes

Tex-specific markers were identified using the FindAllMarkers function, with the heat map of the top ten presented through the DoHeatmap function. Candidate genes for our prognostic risk model were screened through univariate Cox regression, least absolute shrinkage and selection operator (LASSO), and multivariate Cox regression analysis based on two independent cohorts. The risk score for OS samples was calculated as Σ_n_^i^ (Coef_i_ × X_i_). Then, the patients were grouped into high- and low-risk categories according to the median risk score. The survival probability of the two risk groups was compared through the KM curve, while the predictive accuracy of our risk score prognostic model was evaluated by the receiver operating characteristic (ROC) curve. Finally, univariate and multivariate Cox regression analysis were conducted base on the risk score and clinical features.

### Immunofluorescence

To confirm the expression level of candidate genes, we gathered a total of 10 pairs of paraffin-embedded OS tissues and corresponding normal tissues for immunofluorescence and immunohistochemical. This research was approved by the Institutional Review Board of Xijing Hospital, Fourth Military Medical University. And, the informed assent/consent was obtained. Sections were subjected to a 20-min treatment with 0.3% TritonX-100, followed by a 1-h blockade using a 5% BSA blocking solution at ambient temperature. The corresponding primary antibody was introduced to the sections and left to incubate overnight at 4°C. Subsequently, the sections underwent incubation with the secondary antibody (dilution 1:200) at room temperature for an hour, conducted in darkness. Post a 10-min PBS wash, nuclei were stained with DAPI. The employed antibodies include the following: anti-MYC (dilution 1:100, GB13076, Servicibio, China), anti-FCGR2B (dilution 1:500, GB114833-100, Servicibio, China), and anti-CD8 (dilution 1:50, GB12066, Servicibio, China).

### Construction and validation of a nomogram for OS patients

We utilized a nomogram to effectively visualize the Cox proportional hazard model, predicting the 3- and 5-year overall survival rates of OS patients. Furthermore, we employed ROC, calibration, and decision curve to gauge the predictive accuracy of the model.

### Drug susceptibility prediction

In order to predict drug susceptibility in the two Tex risk groups, we employed the "pRRophetic" package. The half-maximal inhibitory concentrations (IC50s) of the drugs were analyzed and presented in a box plot with the Wilcoxon signed-rank test.

### Immunohistochemistry

Following standard protocol, all tissue sections were de-waxed and fixed with an antigen. Subsequently, they underwent blocking and incubation with primary and secondary antibodies (MYC, 1:200, Bioss, bs-4963R; FCGR2B, 1:500, Servicebio, GB114833-100). The slides were then developed using a DAB kit (CWBIO, CW2035S) and counterstained with hematoxylin, before being observed and recorded under a microscope. For analysis, we employed the ImageJ software.

### Statistical analysis

For data analysis, we employed a range of software tools, including R (version 4.2.3), SPSS (version 21.0), and GraphPad Prism (version 8). To compare the data of two groups, we utilized either the t-test or the Mann–Whitney U test, as appropriate. All differences among and between groups were considered statistically significant at *p* values of < 0.05 (*p < 0.05; **p < 0.01; ***p < 0.001).

## Results

### Preprocessing of single-cell RNA sequencing (scRNA-seq) data and annotation of distinct cellular subpopulations

**Fig. 1:**
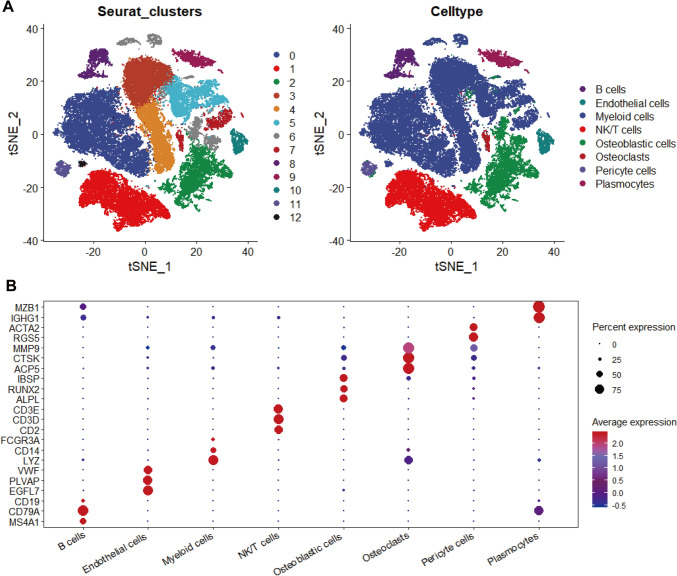
Results of cell clustering in OS and healthy bone tissues. **A** T-SNE plots colored by cell clusters. **B** The expression patterns of marker genes for each cell cluster within the TIME

To discern the compositions of the cells in the immune microenvironment of OS, we conducted scRNA-seq analysis on four healthy bone tissues and six primary OS specimens obtained from patients who had not undergone neoadjuvant chemotherapy. Figure S1 shows the results both pre- and post-filtering of cells and features. Following quality control procedures, 25,264 features and 58,617 cells remained out of the original 33,538 features and 85,212 cells (Figure S2A, B). Figure S2C displays the top 3000 variable features identified. Principal component analysis (Figure S2D) was employed to determine the available dimensions, with the significant components and their corresponding standard deviations illustrated in Figure S3. After successfully mitigating the batch effect by the Harmony package (Figure S4A), the cell clusters from two different sources were effectively integrated (Figure S4B, C). The results of t-distributed Stochastic Neighbor Embedding (tSNE) and their respective annotations are presented in Fig. 1A. The specific marker genes associated with various cell types are outlined in Table S2. Consequently, the cells were classified into eight distinct subclusters: osteoclasts, endothelial cells, plasmocytes, B cells, osteoblastic cells, NK/T cells, pericyte cells, and myeloid cells. The scaled relative expression of these specific marker genes and their respective percentages of expression are visualized in Fig. 1B.

### Identification of CD8^+^ T cells and pseudotime trajectories analysis

**Fig. 2:**
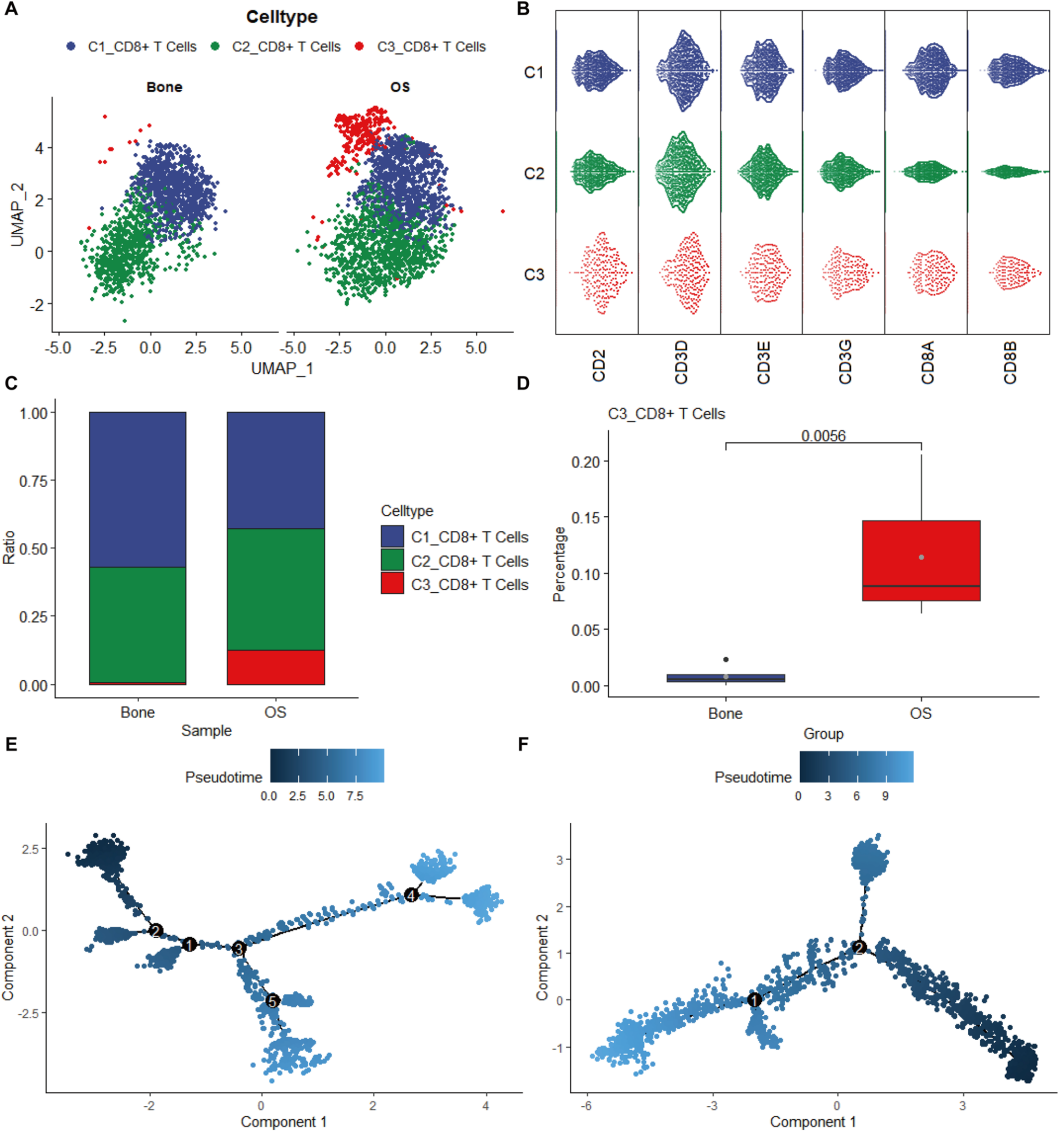
Reclusters of CD8^+^ T cells in OS and healthy bone tissues. **A** UMAP plot shows CD8^+^ T cell subclusters. **B** Bee swarm plot shows the expression of marker genes in CD8^+^ T cell subclusters **C** relative ratio of each cell cluster in OS and bone tissues. **C** The Monocle 3 trajectory plot showing the differentiation of T cell subclusters **D** differences in the infiltration levels of the C3 subpopulation between normal bone tissue and tumor. *P* value was determined by t-test. Pseudotime analysis in normal bone tissue (**E**) and osteosarcoma (**F**)

To elucidate the exhaustion of CD8^+^ T cells in OS, we isolated and classified CD8^+^ T cells into three distinct subtypes. The tSNE results were split by tissue origin (Fig. 2A). The expression patterns of specific marker genes within the CD8^+^ T cell subsets were visually represented using a bee swarm plot (Fig. 2B). All three subtypes exhibited positive expression of CD2, CD3D, CD3E, CD3G, CD8A, and CD8B, thereby validating the accuracy of our cellular annotation. The CD8^+^ T cell subtypes were distributed notably differently between normal bone and OS samples, with a significant elevation of C3 in the latter (*P* value = 0.0056; Fig. 2C, D). The monocle algorithm evinced a marked divergence in the differentiation trajectory of CD8^+^ T cells between normal bone tissue (Fig. 2E) and OS samples (Fig. 2F).

### CD8^+^ T cell exhaustion within normal bone tissue and OS

**Fig. 3:**
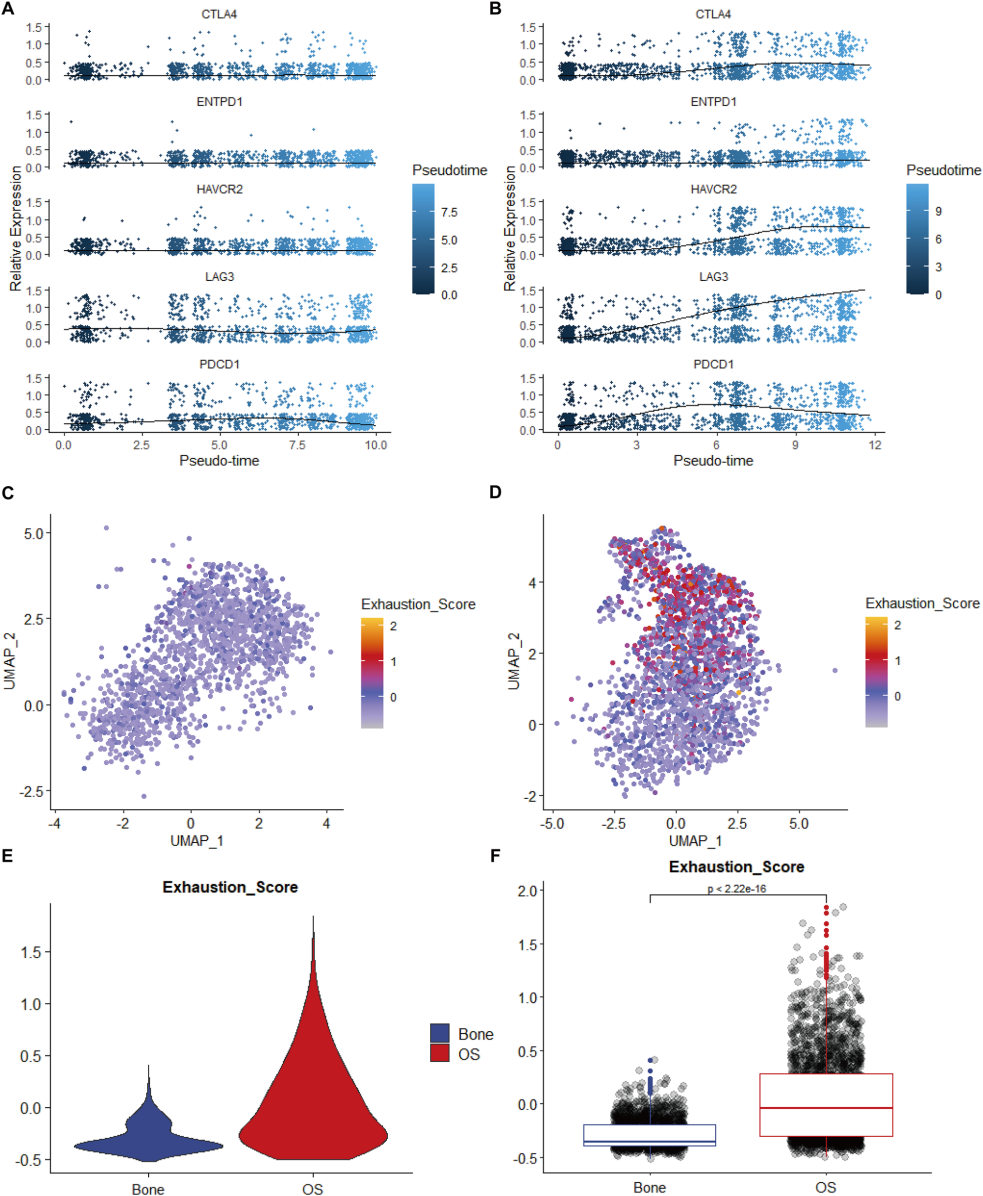
Evaluation of T cell exhaustion in normal bone tissue and osteosarcoma. The expression levels of T cell exhaustion-related genes varying with pseudotime trajectory in both normal bone tissue (**A**) and osteosarcoma (**B**). The exhaustion score in normal bone tissue (**C**) and osteosarcoma (**D**). Violin (**E**) and box (**F**) charts representing the exhaustion score in normal bone tissue and osteosarcoma

**Fig. 4:**
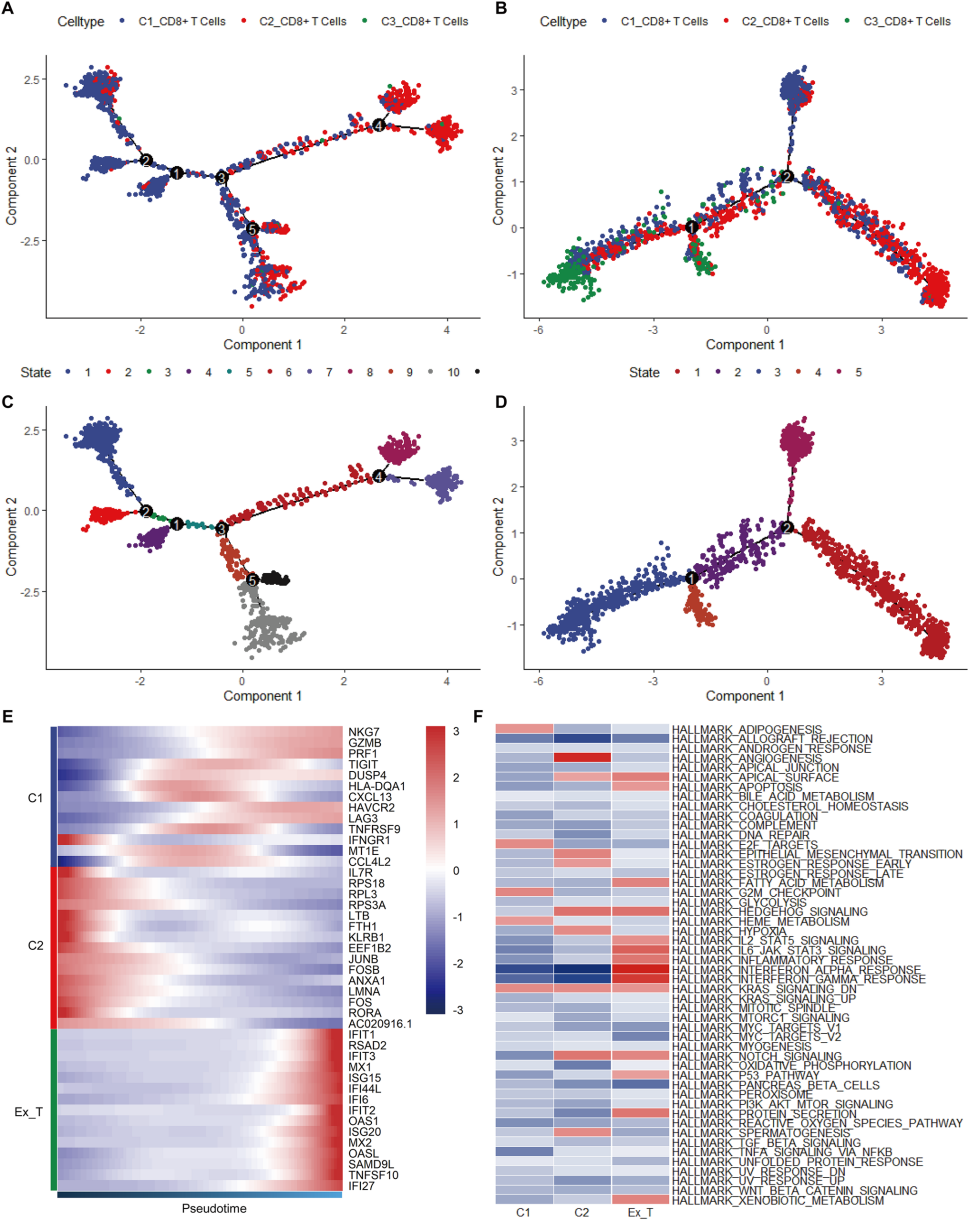
Pseudotime analysis and GSVA. The pseudotime trajectory coloring by cell type in normal bone tissue (**A**) and osteosarcoma (**B**). The pseudotime trajectory coloring by state in normal bone tissue (**C**) and osteosarcoma (**D**). **E** The expression of specific genes in different CD8^+^ T cell subpopulations along the pseudo-time trajectory. **F** The heatmap of GSVA of hallmark pathways between CD8^+^ T cell subpopulations

The plot_genes_in_pseudotime function was used to discern the correlation between the relative gene expression of 13 Tex-associated genes (extracted from the literature) [14] and the pseudotime trajectories in both the normal bone tissue (Figure S5A) and OS (Figure S5B) groups. Five genes—*CTLA4*, *PDCD1*, *ENTPD1*, *LAG3*, and *HAVCR2*—were upregulated towards the end of the trajectory in OS (Fig. 3A) compared with that in normal bone tissue (Fig. 3B). Subsequently, we employed a scoring framework based on these five Tex-associated genes to compute exhaustion scores for both normal bone tissue and OS specimens, which were projected onto the tSNE plots for the same (Fig. 3C, D). Furthermore, we employed violin plots to highlight the disparity in Tex levels between the two groups (Fig. 3E). Remarkably, a significant distinction in Tex levels was observed between the two groups (*P* value < 0.01; Fig. 3F), indicating a greater prevalence of exhausted CD8^+^ T cells in OS. The three distinct CD8^+^ T cell subtypes exhibited divergent paths along the pseudotime trajectories, with C3 CD8^+^ T cells predominantly occupying the terminal position (Fig. 4A, B). Figure 4C, D illustrate the inferred states at different points along the pseudotime continuum. Subsequently, the FindAllMarkers function was employed to identify cluster-specific genes within the CD8^+^ T cell subtypes, and their expression patterns were depicted in a pseudotime heatmap (Fig. 4E), which delineated that the C3 CD8^+^ T cell subtype encompassed a considerable proportion of cells positioned towards the terminus of the trajectory. These results indicate that C3 represented an exhausted population of CD8^+^ T cells that was significantly elevated in OS.

### Gene set variation analysis (GSVA) of CD8^+^ T cells in OS

We employed GSVA to further investigate the molecular signaling pathways involved in CD8^+^ T cell subtypes isolated from OS samples. The GSVA scores for hallmark pathways are recorded in Table S3, and a GSVA heatmap was generated to visualize the total hallmark pathways (Fig. 4F). We found that apoptosis, fatty acid metabolism, xenobiotic metabolism, and interferon (IFN) pathways were highly activated in the exhausted T cells (ExTs) cluster of OS samples. These results provide critical insights into the molecular mechanisms that contribute to Tex and ultimately support the proliferation, invasion, and metastasis of OS cells.

### Screening of Tex-specific prognostic genes and construction of the prognostic model

**Fig. 5:**
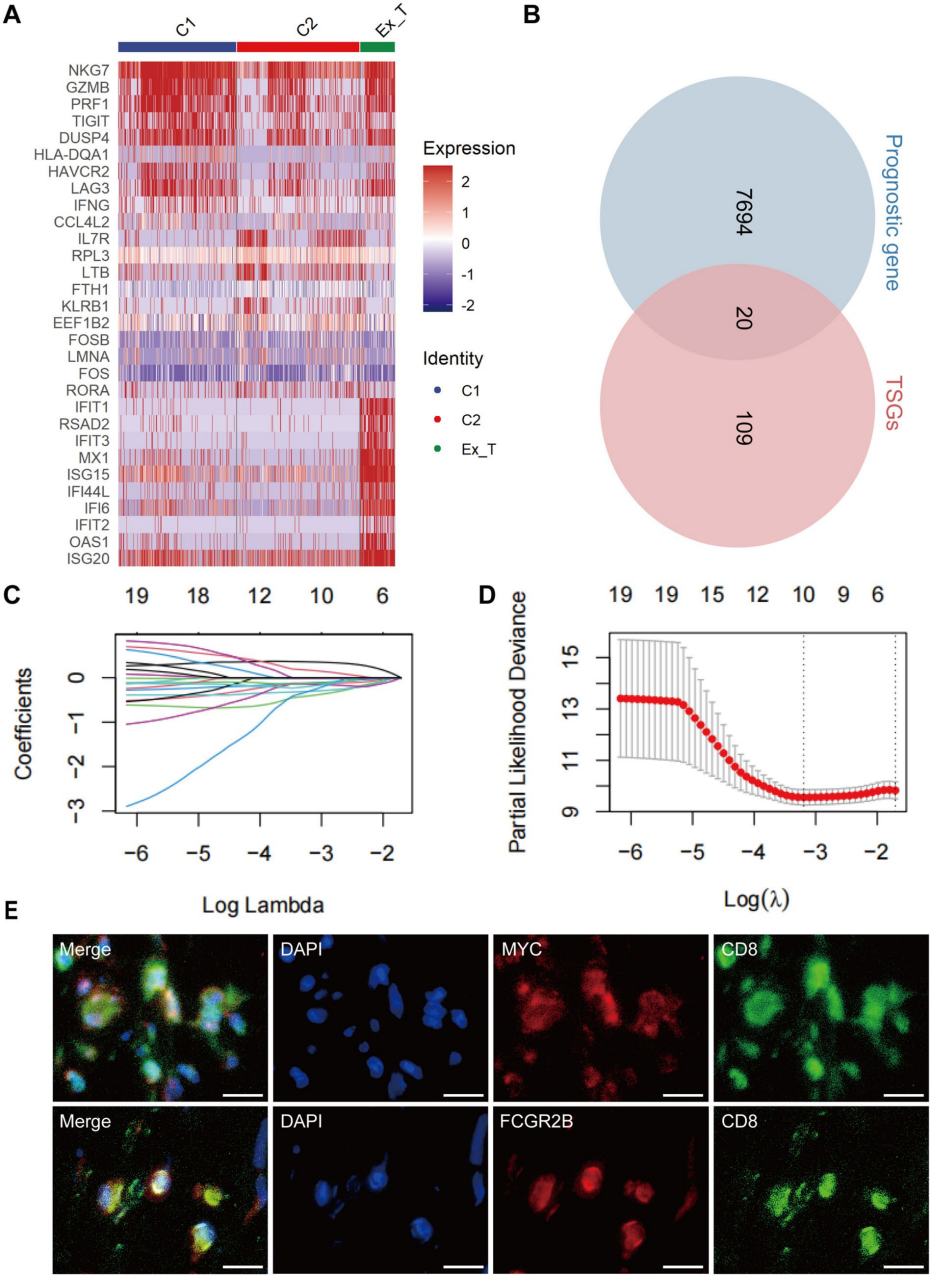
Identification of the Tex-specific genes associated with the prognosis of OS patients. **A** The heatmap of Tex-specific genes. **B** Venn plot of overlapping genes in prognostic gene and Tex-specific genes. **C, D** Lasso regression analysis to further screen candidate genes. **E** Representative micrographs of osteosarcoma sections stained by double immunofluorescence showing the co-expression of MYC, FCGR2B, and CD8 in CD8^+^ T cells. Scale bar indicates 20 μm

**Fig. 6:**
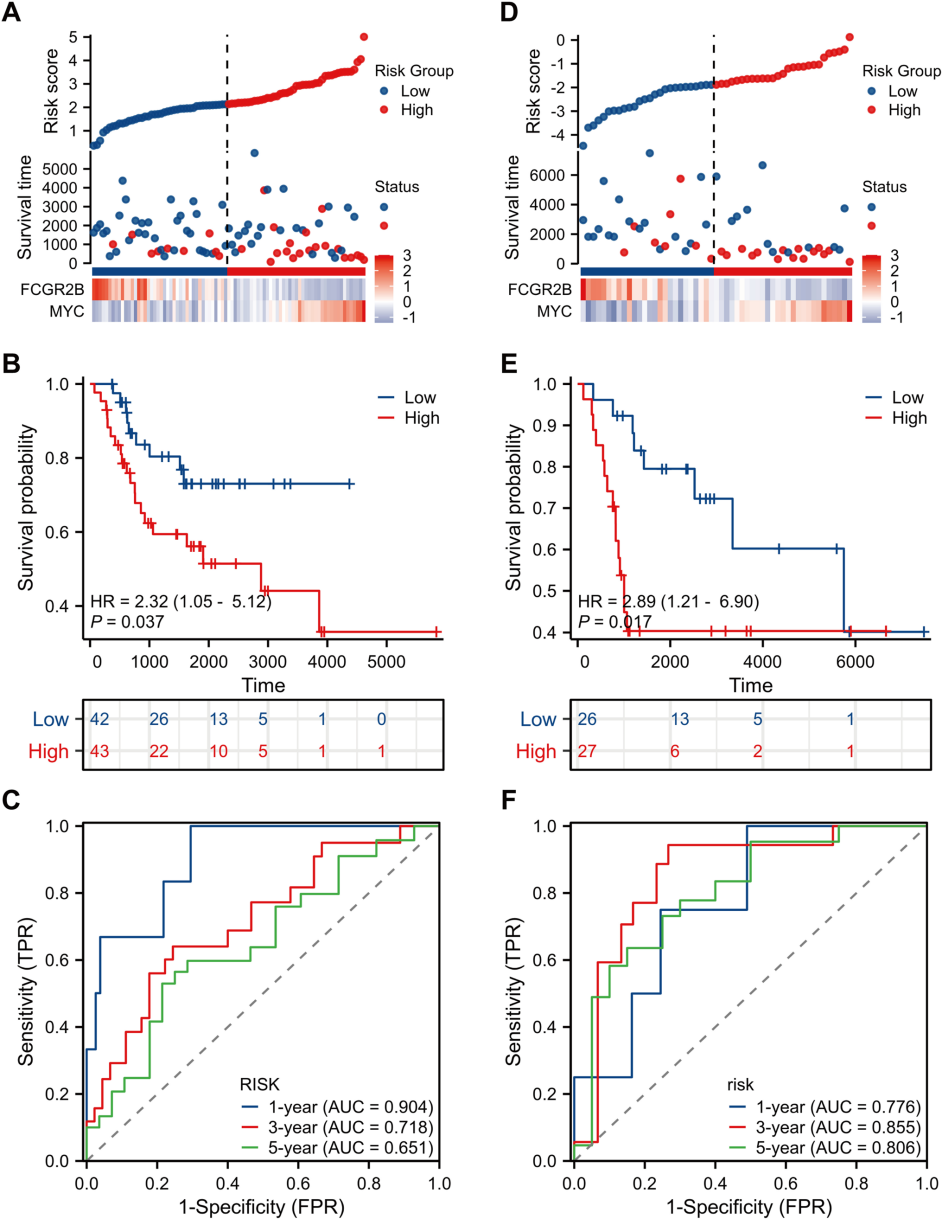
Evaluation and validation of the prognostic model based on Tex-specific genes in both the training and validation cohorts. Risk plot distribution (**A**), KM curve (**B**), and ROC curve (**C**) in the training cohort. Risk plot distribution (**D**), KM curve (**E**), and time-dependent ROC curve (**F**) in the validation cohort

We identified a set of Tex-specific genes using the FindAllMarkers () function (Fig. 5A, Table S4), and screened prognostic genes in two independent cohorts using univariate Cox regression analysis. The Venn diagram shows the 20 promising candidates (Fig. 5B**, **Table S5). Additional screening was conducted using Least Absolute Shrinkage and Selection Operator followed by multivariate Cox regression analysis (Fig. 5C, D), ultimately providing us with two genes, *MYC* and *FCGR2B*. Double immunofluorescence showed the co-expression of these two genes in CD8^+^ T cells (Fig. 5E). The formula for the Tex risk score was defined as 0.569 × *MYC*—0.937 × *FCGR2B*. Using the model, we stratified patients in the TARGET training cohort into low- and high-risk groups, and observed that the low-risk group had a longer survival time than the high-risk group (Fig. 6A). Kaplan–Meier survival analysis revealed a significantly poorer prognosis associated with the high-risk group (*P* value < 0.05; Fig. 6B). The area under the curve (AUC) values at 1, 3, and 5 years was 0.904, 0.718, and 0.651 in the TARGET training cohort, respectively (Fig. 6C). These findings indicated that the Tex risk score accurately predicted the prognosis of OS patients, which was validated in the GSE21257 validation cohort (Fig. 6D–F). The AUC values in validation cohort were 0.776, 0.855, and 0.806 at 1, 3, and 5 years, respectively. Then, we evaluated the clinical prognosis value of the Tex risk score by integrating with clinical characteristics of OS patients in the training cohort. The Tex risk score differed significantly between patients in the M0 and M1 stages, but did not in gender or age, indicating a higher metastatic risk in the high-risk group (Fig. S7A–C). Univariate and multivariate Cox regression analysis revealed that the Tex risk score was an independent prognostic indicator for OS patients (Fig. S7D, E).

### Construction and validation of a prognostic nomogram model

**Fig. 7:**
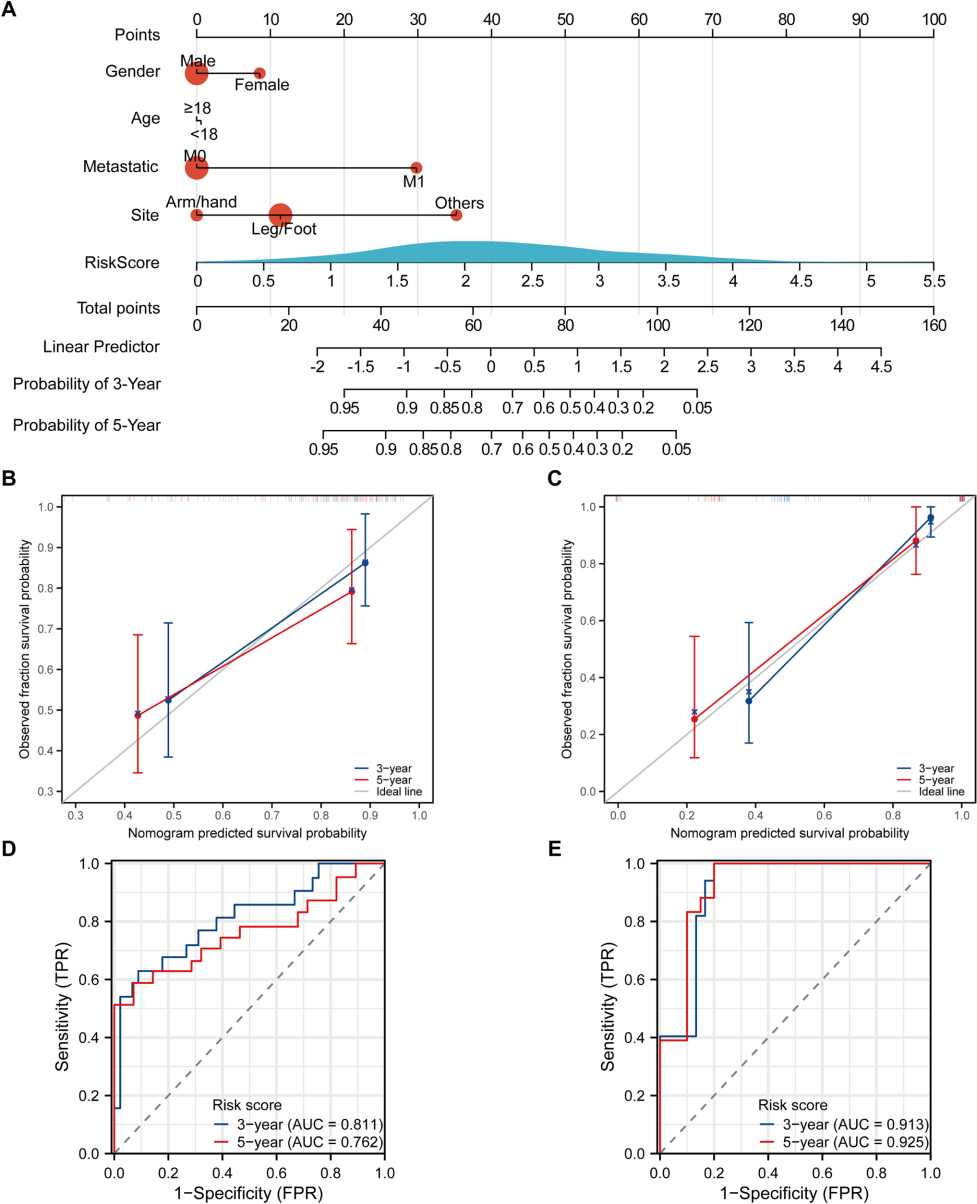
A clinical prognostic nomogram based on Tex-related signatures. **A** A prognostic nomogram to predict 3- and 5-years survival in OS patients. Calibration curve of the nomogram at 3 and 5 years in both the training (**B**) and validation (**C**) cohorts. Receiver operating characteristic curve of the nomogram at 3 and 5 years for the training (**D**) and validation (**E**) datasets

We employed the rms package in R to construct a nomogram for predicting patient survival time. Using Cox regression based on survival time, status, and five clinical characteristics, we achieved a C-index of 0.788 (95% confidence interval: 0.704–0.872; Fig. 7A). To evaluate the prognostic accuracy of our model in the training and validation cohorts, we performed calibration, decision, and receiver operating characteristic curve analyses. Our calibration and decision curves demonstrated excellent predictive ability in both cohorts (Figs. 7B, C, S6). Moreover, the AUC for predicting 3- and 5-year survival were 0.811 and 0.762, respectively, in the training cohort, and 0.913 and 0.925 respectively in the validation cohort, respectively, demonstrating robust prognostic accuracy (Fig. 7D, E).

### Drug susceptibility analysis and immunohistochemistry validation

**Fig. 8:**
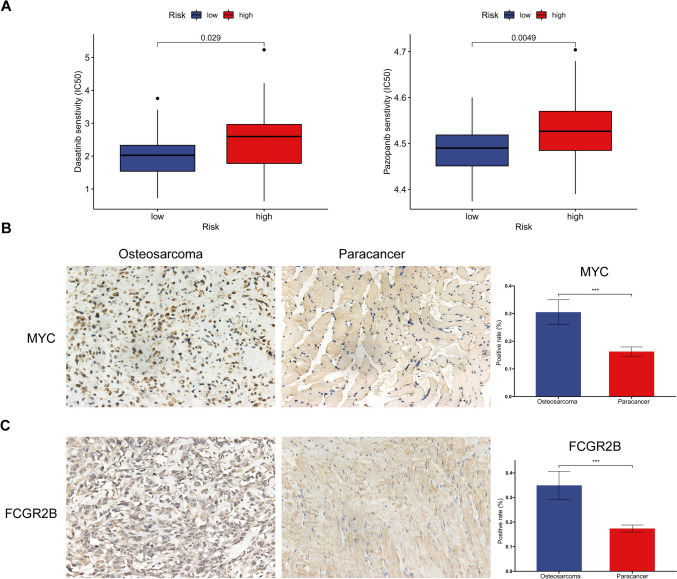
Drugs sensitivity analysis and validation of candidate genes by IHC. **A** Drugs sensitivity analysis in different risk groups. **B, C** The expression levels of candidate genes in osteosarcoma and adjacent tissue using immunohistochemistry. The scale bar in the IHC images represents 100 µm. Statistical significance is denoted by *p < 0.05, **p < 0.01, and ***p < 0.001

The low-risk group demonstrated markedly lower half-maximal inhibitory concentrations for Dasatinib and Pazopanib than the high-risk group (Fig. 8A). Furthermore, we used immunohistochemistry analysis to confirm the expression of *MYC* and *FCGR2B*, both of which were significantly upregulated in OS tissues relative to adjacent normal tissues (Fig. 8B, C).

## Discussion

Tumorigenesis, progression, drug resistance, and immune escape are promoted by defective antitumor responses of immune cells within the highly heterogeneous and complex TIME of OS [15]. T cell exhaustion (Tex) has been demonstrated to exert immunosuppressive effects in the TIME and constrain T cell-based immunotherapies [16]. However, its specific role in the context of OS remains unclear. Comprehensively understanding Tex within the immune microenvironment of OS holds the key to overcoming it and enhancing clinical checkpoint blockade immunotherapies. Targeting Tex has emerged as a promising approach in the field of cancer immunotherapy [17]. Because targeting co-inhibitory checkpoints like PD-1 and CTLA-4 alone is insufficient to fully restore T cell function, identifying other molecules involved in Tex is imperative. The advent of single-cell sequencing technologies has enabled interpretation of the TIME of OS at the resolution of individual cell level. This approach facilitates identification of rare subpopulations within the TIME and exploration of their pro-tumorigenic functions as well as phenotypic plasticity.

In the current study, we performed single-cell clustering analysis in the single-cell RNA transcriptome data obtained from OS samples and normal bone tissues, and successfully identified eight distinct cell types within the integrated dataset. To unravel the heterogeneity of CD8^+^ T cells within the TIME of OS, a secondary clustering analysis was conducted to extract and categorize CD8^+^ T cells into three distinct subtypes in both the OS and healthy bone tissue groups. We found a substantially higher infiltration of C3 cluster cells in the OS samples. By assessing the expression levels of Tex marker genes across all cells, we observed varying degrees of Tex in the three CD8^+^ T cell clusters in OS compared with healthy bone tissue. Enrichment of Tex in the TIME has been associated with poor prognosis in various tumors. Shen et al. demonstrated that patients with high Tex in chronic obstructive airway disease exhibited poorer prognosis, whereas patients with low Tex responded better to chemotherapy and immunotherapy [18]. Similarly, the Tex risk score was significantly associated with survival prognosis in esophageal adenocarcinoma [19]. Subsequently, we conducted pseudotime analysis on CD8^+^ T cells, revealing that the C3 cluster occupied the terminal state in the pseudotime trajectory. This finding confirmed the functional exhaustion characteristics of the C3 CD8^+^ T cell cluster. Furthermore, we examined the expression patterns of Tex marker genes along the pseudotime trajectory. In comparison to healthy bone tissue, we found that the expression of various Tex marker genes (*PDCD1*, *CTLA4*, *LAG3*, *ENTPD1*, *HAVCR2*) gradually increased with pseudotime in OS. PD-1, a pivotal co-inhibitory receptor on activated T cells, interacts with overexpressed programmed cell death ligand 1 (PD-L1) on cancer cells, tumor-infiltrating lymphocytes, and stromal cells, detrimentally affecting the cytotoxicity of CD8^+^ T cells and consequently mediating immunosuppressive responses [20]. The upregulation of PD-L1 and PD-1 was shown to be correlated with adverse prognosis as well as relapse or metastasis in OS patients [21]. During tumor treatment, blockade of the PD-1 pathway can reactivate exhausted CD8^+^ T cells by reprogramming metabolism, promoting proliferation, and enhancing the expression of effector molecules [22]. However, in comparison to other tumors, OS is associated with low PD-L1 expression, poor immune infiltration, and limited response to checkpoint blockade [23]. CTLA-4 is an inhibitory receptor predominantly expressed on T cells that binds to CD80/CD86 on antigen-presenting cells, leading to impaired T cell function. In vivo studies have demonstrated that OS patients exhibit enhanced anti-tumor activity of cytotoxic T cells following treatment with CTLA-4 antibodies, revealing the potential of CTLA-4 inhibitors in the treatment of OS [24]. LAG-3 is highly expressed by tumor-infiltrating lymphocytes in cancer and represents a non-immunoreceptor inhibitory receptor with a tyrosine-based inhibitory motif. It negatively regulates the cell cycle and cellular functions through the KIEELE motif [25]. ENTPD1 (CD39) is a cell surface-expressed enzyme that hydrolyzes extracellular ATP. The binding of extracellular ATP to P2X receptors on T cells induces cytokine production and proliferation. Therefore, by hydrolyzing extracellular ATP, ENTPD1 impairs the function of effector T cells and mediates Tex [26]. HAVCR2 can interact with PD-1 and galectin-9, thereby regulating Tex and the efficacy of immunotherapy [27]. In conclusion, we ultimately identified five co-inhibitory molecules that mediate Tex in OS, thus providing valuable insights and novel immunotherapy targets.

The functional alterations to CD8^+^ T cell subpopulations in OS were investigated using GSVA. Apoptosis, fatty acid metabolism, xenobiotic metabolism, and the IFN pathway were found to be significantly activated in ExTs in OS. The microenvironment of OS is primarily hypoxic and acidic. Tumor cells predominantly depend on aerobic glycolysis and fatty acid metabolism to meet their energy demands, a metabolic strategy shared by highly efficient effector T cells [28]. Consequently, the competition for glucose as well as other fuel sources, such as fatty acids and oxygen, may detrimentally affect the proliferation and activation of effector T cells, culminating in a state of exhaustion within the TIME. Simultaneously, the immunosuppressive metabolic by-products generated by the tumor itself may impede the functionality of T cells. The PD-1 signaling pathway is intricately connected to T cell metabolic pathways, and the impact of PD-1 blockade on the metabolism of ExTs has been investigated. The PD-1 signaling pathway inhibits the activation of protein kinase B, thereby suppressing the activity of mammalian target of rapamycin and eventually inhibiting T cell glycolysis [29]. Blocking the PD-1 signaling pathway reactivates the synthetic metabolism of ExTs and enhances glucose uptake in a mammalian target of rapamycin-dependent manner, which may contribute to the improvement of tumor-infiltrating lymphocyte function and tumor regression [30]. These findings suggest that cellular metabolic reprogramming may represent a crucial strategy for Tex reversal during immunotherapy. We also noted a pronounced activation of the IFN pathway in ExTs in OS. IFN-α/β are crucial pro-inflammatory cytokines that exhibit dual roles in tumors. They can suppress tumor growth by inducing anti-tumor activity within the immune system and activating innate immune cells [31]. The IFN-α/β signaling pathway is indispensable for T cell development and the generation of effector and memory T cells [32]. However, during cancer, IFN-α/β levels may rise, inducing the expression of PD-L1, a negative regulator of the immune system [33]. IFN-α/β can also promote the functional exhaustion of activated T cells through Fas/FasL-mediated T cell death [34]. Highly activated IFN-α/β signaling can also promote the terminal exhaustion of functional T cells by interfering with the transcription factor T cell factor-1, thus antagonizing the reservoir of progenitor ExTs [35]. Targeting these molecules or their corresponding receptors represents a promising strategy to reverse the impact of the tumor microenvironment on T cell function.

To uncover the prognostic role of Tex in OS, we assessed the prognostic value of Tex-specific genes using two independent OS bulk RNA-seq datasets. Subsequently, two Tex-specific biomarkers (*MYC* and *FCGR2B*) were selected to construct a Tex risk model. *MYC*, an oncogene encoding a nuclear phosphoprotein, plays a role in cell cycle progression, apoptosis, and cellular transformation. Amplification of this gene is frequently observed in many human cancers [36]. Previous studies have demonstrated that c-Myc is necessary for the S100A9-induced upregulation of PD-1/PD-L1 [37]. The overexpression of *MYC* induces the expression of CD47 and PD-L1 in tumor cells, allowing them to evade immune surveillance. Therapies aimed at inhibiting *MYC* expression and activity may potentially restore immune responses against human cancers [38]. The hypoxic state of the tumor microenvironment induces mitochondrial defects and promotes Tex in the TIME via the *MYC* regulatory pathway [39]. FCGR2B, a low-affinity receptor in the Fc region of the immunoglobulin gamma complex, is known to participate in the phagocytosis of immune complexes. Previously, FCGR2B was believed to be expressed exclusively on B cells and innate immune cells, but recent studies have demonstrated a significant upregulation of FCGR2B on tumor-infiltrating effector CD8^+^ T cells, suggesting that it may serve as a novel T cell checkpoint in anti-tumor immunity [40]. Morris et al. discovered that genetic defects in FCGR2B can enhance the tumor-infiltrating CD8^+^ T cell response and cause the tumor volume to decrease [41]. The Tex risk model constructed using *MYC* and *FCGR2B* accurately predicted patient prognosis in both the training and validation cohorts. Furthermore, individuals with OS who exhibit a low risk of Tex demonstrated heightened responsiveness to tyrosine kinase inhibitors like Dasatinib and Pazopanib. Protein tyrosine kinases are instrumental mediators of signal transduction, facilitating phospho-transfer onto tyrosine residues. The aberrant expression of tyrosine kinases is intricately associated with invasiveness, metastasis, and angiogenesis of tumors [42]. Consequently, diverse tyrosine kinase inhibitors have been used to treat various solid malignancies, significantly enhancing the survival and quality of life of patients [43]. Hence, we posit that the Tex risk score carries promising potential to predict the effectiveness of targeted therapeutic interventions.

To the best of our knowledge, this is the first investigation of the role of Tex in the TIME of OS patients. Nonetheless, we must acknowledge certain limitations of this study. Firstly, the inherent heterogeneity among OS patients may limit the generalizability of the findings obtained through single-cell RNA-seq studies. These results must be validated in large-scale cohorts to ensure their broader applicability. Secondly, the limited number of single-cell OS samples resulted in a relatively small population of ExTs, which restricts the depth of our understanding regarding their precise functions within the TIME of OS. Finally, the molecular mechanisms that promote Tex in the TIME of OS via *MYC* and *FCGR2B* necessitate further investigation in the future.

## Conclusions

This study explored the role of T cell exhaustion in the immune microenvironment of OS. The single-cell sequencing data revealed a notable increase in functionally exhausted T cells within OS samples, accompanied by the upregulation of exhaustion marker genes, such as *PDCD1*, *CTLA4*, *LAG3*, *ENTPD1*, and *HAVCR2*. Simultaneously, we observed a heightened activation of apoptosis, fatty acid metabolism, xenobiotic metabolism, and the IFN pathway within the ExTs population in OS samples. Finally, we developed a prognostic model based on two Tex-related signatures that accurately predicted the clinical outcomes of OS patients. These findings offer novel perspectives for clinical decision-making and the formulation of treatment strategies in the context of OS.

### Supplementary Information

Below is the link to the electronic supplementary material.

Supplementary file1 (TIF 23512 kb)

Supplementary file2 (TIF 18784 kb)

Supplementary file3 (TIF 23416 kb)

Supplementary file4 (TIF 19938 kb)

Supplementary file5 (TIF 19922 kb)

Supplementary file6 (TIF 5919 kb)

Supplementary file7 (DOCX 25 kb)

Supplementary file8 (XLSX 998 kb)

Supplementary file9 (XLSX 12 kb)

Supplementary file10 (XLSX 75 kb)

Supplementary file11 (XLSX 11 kb)

Supplementary file12 (TIF 13142 kb)

## Data Availability

All data are freely available from public databases, and other necessary and reasonable information can be obtained from the corresponding author.

## Abbreviations

AUC: Area under the curve
Bulk RNA-Seq: Bulk RNA sequencing
CTLA-4: Cytotoxic T lymphocyte-associated protein-4
ExTs: Exhausted T cells
GEO: Gene Expression Omnibus
GSVA: Gene set variation analysis
IC50s: Half-maximal inhibitory concentrations
IFN: Interferon
IHC: Immunohistochemistry
LAG-3: Lymphocyte-activation gene-3
LASSO: Least Absolute Shrinkage and Selection Operator regression
OS: Osteosarcoma
PCA: Principal component analysis
PD-1: Programmed cell death-1
PD-L1: Programmed cell death ligand-1
ROC: Receiver operating characteristic
scRNA-Seq: Single-cell RNA sequencing
Tex: T cell exhaustion
TME: Tumor microenvironment
tSNE: T-Distributed Stochastic Neighbour Embedding
