# Supplementary figures and images for: Unraveling T cell exhaustion in the immune microenvironment of osteosarcoma via single-cell RNA transcriptome
Source: Cancer Immunol Immunother. 2024 Jan 27;73(2):35. doi: 10.1007/s00262-023-03585-2 (PMC10821851; doi:10.1007/s00262-023-03585-2)

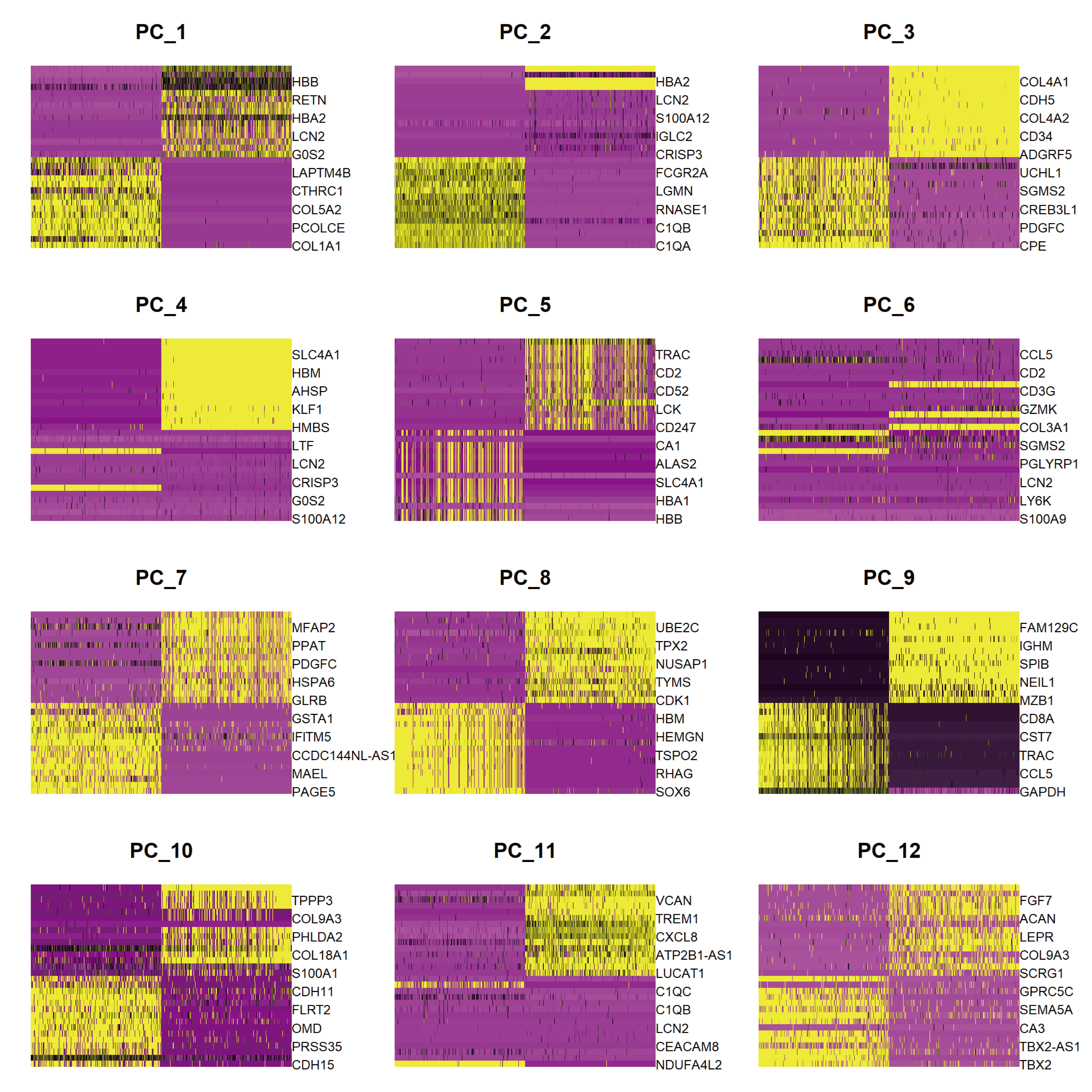

Supplement: Supplementary file 3 — Supplementary file3 (TIF 23416 kb) [file 262_2023_3585_MOESM3_ESM.tif]

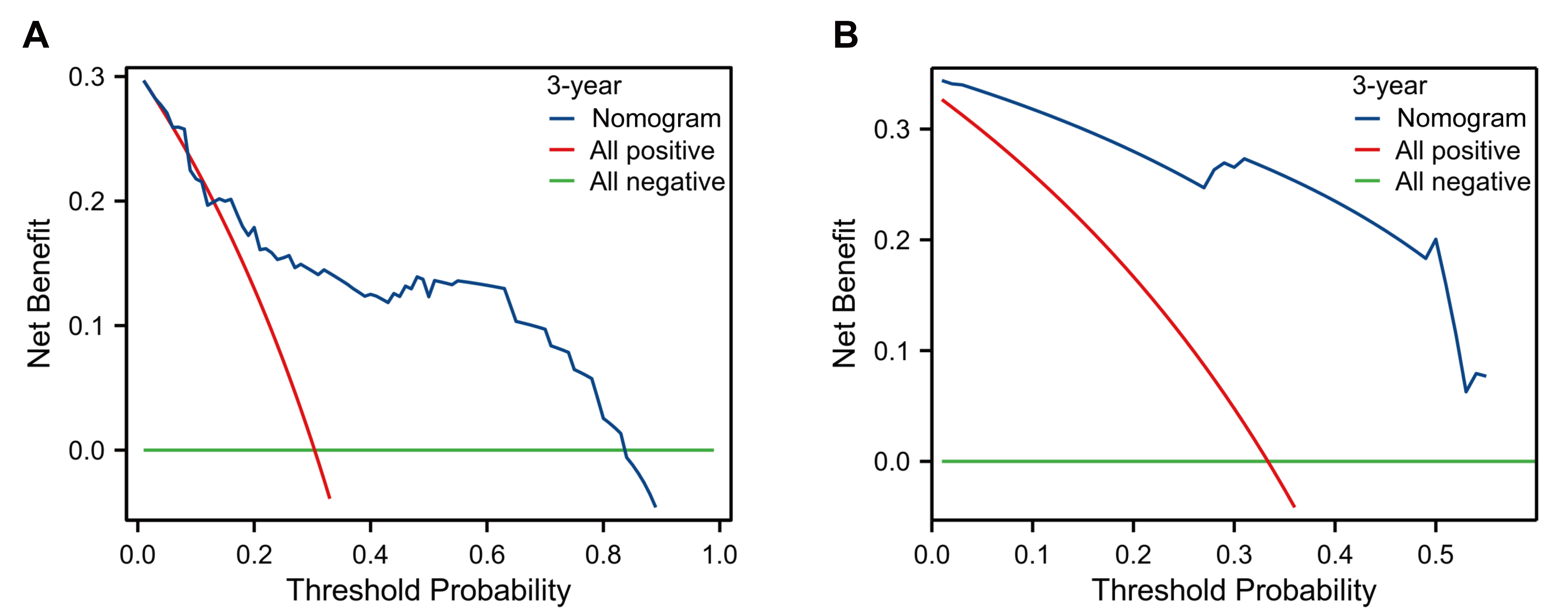

Supplement: Supplementary file 6 — Supplementary file6 (TIF 5919 kb) [file 262_2023_3585_MOESM6_ESM.tif]
