# Supplementary material for: Unraveling T cell exhaustion in the immune microenvironment of osteosarcoma via single-cell RNA transcriptome
Source: Cancer Immunol Immunother. 2024 Jan 27;73(2):35. doi: 10.1007/s00262-023-03585-2 (PMC10821851; doi:10.1007/s00262-023-03585-2)
Supplement: Supplementary file 7 — Supplementary file7 (DOCX 25 kb) [file 262_2023_3585_MOESM7_ESM.docx]

**Table S1.** Clinical characteristics of patients with osteosarcoma in the training set and validation set.

|  |  | TCGA training set  (n = 85) | GSE21257 validation set (n = 53) |
| --- | --- | --- | --- |
| Age, n (%) | <18 | 67(78.8) | 35(66.0) |
|  | ≥18 | 18(21.2) | 18(34.0) |
| Gender, n (%) | Female | 37(43.5) | 19(35.8) |
|  | Male | 48(56.5) | 34(64.2) |
| Metastasis | M0 | 64(75.3) | 34(64.2) |
|  | M1 | 21(24.7) | 19(35.8) |
| Tumor site | Leg/Foot | 77(90.6) | 44(83.0) |
|  | Arm/hand | 6(7.1) | 8(15.1) |
|  | Others | 2(2.3) | 1(1.9 |
| Status, n (%) | Alive | 56(65.9) | 30(56.6) |
|  | Dead | 29(34.1) | 23(43.4) |

**Table S2.** The marker genes characterizing different cell types within osteosarcoma and normal bone samples.

**Table S3.** The GSVA score for HALLMARK pathway of Each CD8+ T cell subclusters.

**Table S4.** The specific genes of exhausted T cells.

**Table S5.** Identification of candidate genes for constructing prognostic models by univariate Cox regression analysis.

**Figure S1.** Preprocessing of single-cell sequencing data. The violin plot depicts the distribution of unique gene features, total gene expression, and the proportion of mitochondrial genes before (A) and after (B) the data preprocessing step.

**Figure S2.** Preprocess of filtered cells. (A, B) Scatter plots visualize the correlations between mitochondrial genes, gene counts, and gene features. A plot (C) showcasing the top ten highly variable genes, with the X-axis representing the average expression and the Y-axis representing the standardized variance. (D) Principal component analysis (PCA) of all samples.

**Figure S3.** A heatmap focusing on a specific principal component, where cells and genes are categorized based on their respective principal component scores.

**Figure S4.** Removal of batch effects by Harmony package. (A) The convergence plot of the clustering objective function. Comparation of the effects of integration before (B) and after (C) removal of batch effects.

**Figure S5.** T cells exhaustion related genes expression patterns along the entire pseudotime in both the normal bone (A) and OS (B) groups.

**Figure S6.** Decision curves for the training (A) and validation (B) datasets.

**Figure S7.** Univariate and multivariate Cox regression analysis of combining the risk score and clinical features. (A-C) The violin plots show the relationship between risk score and various clinical features. Univariate (D) and multivariate (E) Cox regression analysis to assess the prognostic significance of the risk score in combination with clinical characteristics.
